# Enhancement of recycled aggregate concrete properties through the incorporation of nanosilica and natural fibers

**DOI:** 10.1016/j.heliyon.2024.e39924

**Published:** 2024-11-06

**Authors:** Sattawat Haruehansapong, Prachoom Khamput, Pruchaya Yoddumrong, Wunchock Kroehong, Vichayaphong Thuadao, Akkadath Abdulmatin, Wachirakorn Senawang, Tawich Pulngern

**Affiliations:** a Department of Civil Engineering, Faculty of Engineering and Architecture, Uthenthawai Campus, Rajamangala University of Technology Tawan-ok, Bangkok, 10330, Thailand; b Department of Civil Engineering, Faculty of Engineering, Rajamangala University of Technology Thanyaburi, Pathumthani, 12110, Thailand; c Department of Civil Engineering, Faculty of Engineering, Princess of Naradhiwas University, Narathiwat, 96000, Thailand; d Department of Civil Engineering, Faculty of Engineering, Nakhon Phanom University, Nakhon Phanom, 48000, Thailand; e Department of Civil Engineering, Faculty of Engineering, King Mongkut's University of Technology Thonburi, Bangkok, 10140, Thailand

**Keywords:** Recycled aggregate concrete, Sisal fiber, Palm fiber, Nanosilica, Sustainability

## Abstract

This study introduces an innovative approach to enhancing recycled aggregate concrete (RAC) by incorporating nanosilica (NS) and natural fibers (NF), specifically sisal fiber (SF) and palm fiber (PF). This novel combination aims to overcome the inherent limitations of RAC, such as reduced strength and durability, while promoting sustainability in construction. The research focuses on evaluating the mechanical properties of RAC, including compressive and flexural strengths, through the integration of NS and NF. Our findings reveal that NS significantly improves the microstructure of RAC by enhancing the interface transition zone (ITZ) and filling nanovoids, resulting in a denser and more durable concrete matrix. Specifically, the addition of 3 % NS increased the compressive strength of RAC by up to 22.5 % and the flexural strength by up to 25.6 % at a 100 % replacement ratio of recycled aggregate. The addition of NF, treated to withstand the alkaline environment of concrete, further strengthens the RAC by providing a bridging effect that enhances flexural strength by up to 46.7 %. This work not only advances the performance of recycled concrete but also aligns with the broader goal of environmental sustainability by utilizing waste materials and reducing the carbon footprint of concrete production. The findings have the potential to influence future construction practices, encouraging the adoption of more durable and eco-friendly building materials.

## 1 Introduction

In the quest for sustainable construction methodologies, the enhancement of recycled aggregate concrete (RAC) properties through innovative interventions has emerged as a critical area of research. The use of RAC, which incorporates recycled aggregates from demolished concrete, offers a promising avenue for environmental conservation and resource efficiency in the construction sector. Construction waste, which includes materials such as concrete, bricks, wood, and metals, represents a significant environmental challenge due to its large volume and potential impact on landfills. Utilizing this waste in new concrete production not only mitigates the environmental burden but also contributes to resource conservation by reducing the demand for natural aggregates. This approach aligns with the principles of a circular economy, where materials are reused and recycled, thus closing the loop of resource utilization. However, the inherent limitations of RAC, primarily its reduced strength and durability compared to conventional concrete, necessitate the exploration of methods to bolster its properties. In recent years, additional techniques such as carbonation and compression casting have been developed to further improve the performance of RAC. Carbonation, for instance, enhances the strength and reduces the porosity of RAC through a reaction with carbon dioxide, while compression casting decreases the total porosity and pore ratio in the interface transition zones (ITZs), leading to significant improvements in mechanical properties.

The incorporation of nanosilica (NS) and natural fiber (NF) into RAC presents a novel approach to address these limitations. NS is nanoscale silicon dioxide, which has been increasingly recognized for its potential to enhance the mechanical properties and durability of concrete. Jo et al. (2007) [1] and Qing et al. (2007) [2] showed that adding NS to concrete improves its microstructure and accelerates the pozzolanic reactions, making it stronger and increasing its longevity. Additionally, Senff et al. (2012) [3] demonstrated that NS could increase the durability of cementitious composites by reducing their water absorption and introducing pores. Mukharjee et al. (2014) [4] investigated the effects of colloidal NS incorporation on the performance characteristics of concrete entirely composed of RAC. The experimental findings indicated that the compressive and tensile strengths were enhanced by the addition of NS. Ehsani et al. (2016) [5] demonstrated that the incorporation of NS into cement paste and concrete enhanced strength development and accelerated the reactivity of early age cement. Liang et al. (2022) [6] examined whether the properties of RAC could be improved by combining NS particles with a newly proposed physical compression casting method. In the interface transition zones (ITZs), the total porosity and pore ratio of mortar could be lowered by compression casting or a combination of compression casting and NS particle treatment.

Simultaneously, investigations into the practical use of NF as a reinforcement substitute in concrete have been conducted. In addition to being environmentally sustainable, NF, such as hemp, sisal fiber (SF), and palm fiber (PF), enhance the mechanical properties of concrete. Studies by Toledo Filho et al. (2000) [7] and Marar et al. (2012) [8] demonstrated that the incorporation of NF enhanced the tensile strength and fracture toughness of concrete. These fibers acted as bridges across cracks, improving the post-cracking behavior and energy absorption capacity of the composite material. Hussein et al. (2021) [9] examined the effect of fibers and nanomaterials on the fresh and hardened properties of the produced combinations. They focused on the microstructure, electrical resistivity, density, workability, and mechanical and durability performances. Wang et al. (2023) [10] incorporated basalt and SFs into lightweight roadbed foam concrete and investigated the enhancement of its mechanical properties and durability. The inclusion of mixed NF substantially improved the freeze–thaw resistance and performance stability under varying conditions, according to durability tests. Chen et al. (2023) [11] concluded that the incorporation of NF into concrete increases its tensile strength and decreases its fracture rate. Although adding fibers to concrete improves the tension distribution and consequently enhances its stability, it also leads to a decrease in the compressive strength. The incorporation of fibers into RAC, such as basalt fibers, has been shown to influence the compressive stress-strain relationship, although it also introduces variability in mechanical properties. Understanding this variability is key to optimizing the performance of fiber-reinforced RAC for structural applications [12]. The application of vacuum-based pozzolana slurry encrusted RCA has shown promising results in enhancing the mechanical performance and durability of RAC. The inclusion of basalt fibers further contributes to this enhancement, providing significant improvements in compressive, tensile, and flexural strengths [13]. In addition, the combination of pozzolana slurry treated RCA and BF in FRAC formulations has been found to significantly enhance microstructure quality and mechanical properties, making it suitable for structural applications [14].

Although the individual benefits of NS and NF in enhancing concrete properties are well documented, their combined influence on RAC remains relatively unexplored. This study aimed to fill this research gap by investigating the synergistic effects of NS and NF on RAC. The idea is that the microlevel enhancements brought about by NS and the macrolevel reinforcement offered by NF can significantly improve the strength and durability of RAC. One study by Li et al. (2004) [15] examined the changes in the microstructure of cementitious composites with NS. Another study by Silva et al. (2005) [16] examined the mechanical properties of fiber-reinforced concrete. Both studies showed that this combined approach may be useful. Research by Medina et al. (2012) [17] on the use of recycled fibers in concrete further underscored the potential of combining recycled materials with NF for improved performance. To determine how different amounts of NS and NF affect the properties of RAC, this study used a thorough experimental method that included both mechanical testing and microstructural analysis. The methodology was designed to build upon the findings of previous studies, such as those by Peimin et al. (2022) [18], who explored the mechanical properties of RAC with nanomaterials, and Aliakbar et al. (2022) [19], who investigated the durability of NF-reinforced concrete. Adetukasi et al. (2021) [20] investigated plain concrete as a control, fiber-reinforced concrete (without NS), and fiber-reinforced concrete containing NS, and NS and fiber reinforcement were found to improve the concrete performance, in that the mechanical properties were significantly enhanced.

The implications of this research are far-reaching and extend beyond the realm of material science to impact sustainable construction practices. Enhancing the properties of RAC by incorporating NS and NF not only promotes the use of recycled materials, but also contributes to the development of more durable and sustainable construction materials. This aligns with the goal of reducing the environmental footprint of the construction industry, as emphasized in studies by Pacheco-Torgal and Jalali (2011) [21] and Medina et al. (2012) [22]. Furthermore, Ogunbode et al. (2017) [23] and Abdalla et al. (2023) [24] revealed that the utilization of NF as a fibrous material in the manufacturing of environmentally friendly and technically sustainable concrete exhibits considerable potential for the foreseeable future. The optimization of mix designs for RAC is crucial for enhancing its mechanical properties and sustainability. Recent advancements, such as the development of agile and scalable frameworks for mix design optimization, have shown significant potential in incorporating recycled aggregates from precast rejects, leading to more efficient and eco-friendly concrete formulations [25].

This study represents a significant step toward the development of high-performance and sustainable construction materials. By bridging the gap in the current understanding of the combined effects of NS and NF in RAC, this study aimed to contribute to the advancement of sustainable construction technologies and practices, and to identify the composite enhancement mechanism of NS and NF (PF and SF) in RAC. This study investigated the mechanical properties of RAC by utilizing the RAC replacement ratio and PF, SF, and NS contents and examined the mechanical properties of RAC, such as its flexural and compressive strengths, at 7 and 28 d. In addition, the microstructures of the samples were examined at 28 d using scanning electron microscopy (SEM) and X-ray diffraction (XRD). The outcomes of this research can significantly influence building codes and practices, leading to wider acceptance and use of RAC in mainstream construction. The exploration of these innovative materials in RAC also opens new possibilities for research and development in the field of construction materials, potentially leading to further breakthroughs in sustainable construction methodologies.

## 2 Experimental details

### 2.1 Materials

Type I ordinary Portland cement (PC) mainly comprises clay and limestone. The cement used primarily contained oxides such as calcium oxide (CaO), silica (SiO_2_), alumina (Al_2_O_3_), and ferric oxide (Fe_2_O_3_). The cement content of these four oxides exceeded 90 % and their average particle size was 15,000 nm. River sand was graded according to the ASTM C778 standard [26]. Evonik Industries produces NS in Germany. Market NS products typically contain SiO_2_ in excess of 99.8 %. The physical properties of NS are conspicuously apparent in the shape of ultrafine white dust with a particle size of approximately 12 nm. The manufacturer's data indicates that the specified surface area of the NS products is 200 m^2^/g. Table 1 lists the physical properties of NS and PC, as provided by the manufacturer.

**Table 1:** Physical properties of PC and NS.

Items	Chemical Composition (%)	
	Ordinary Portland cement type I (PC)	Nanosilica (NS)
**SiO**_**2**_	22	99.8
**Al**_**2**_**O**_**3**_	6.6	–
**Fe**_**2**_**O**_**3**_	2.8	–
**CaO**	60.1	–
**MgO**	3.3	–
**Diameter (nm)**	15,000	12

SF is an exceptionally simple-to-grow NF that has been extensively utilized. Previously, this hard fiber was employed in the production of twines, ropes, and fiber cores for elevator steel wire cables. It is obtained from the sisal plant in Prachuapkhirikhan Province. To facilitate the deposition of nanoparticles on the fiber surface, it is usually necessary to clear impurities from the fiber first [27]. Alkaline treatment is the commonly selected pre-treatment method. Therefore, SF was treated with a 5 % solution of sodium hydroxide solution (NaOH) (0.1 concentration) for 20 min and dried. Subsequently, it was washed with 1 % acetic acid to remove any excess NaOH. After curing at room temperature, the SF was incorporated into the concrete. The average NF length was 20 mm.

**Fig. 1:**
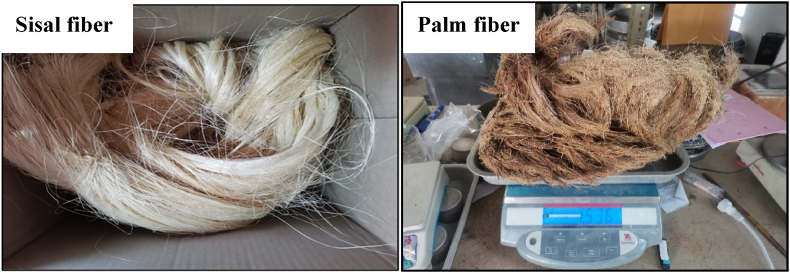
Sisal fiber and palm fiber.

The PFs utilized in this study were by-products of empty fruit bunches from the Samut Prakan Province palm oil production industry. Empty fruit bunch fibers are the fiber residues remaining after the removal of the fruit and processing of oil from the fruit bunch. After a 2-h boiling process, empty bunches of fruit were washed with water at a pH of approximately 7. The moisture in the raw fibers was extracted via sun drying for 6 h. The oil PFs were subsequently divided and trimmed using a processing machine. To alkalize the oil PFs, they were soaked in a 2 % NaOH solution for 24 h before drying in an oven [28,29]. Fig. 1 shows the appearance of SF and PF. The supplier descriptions of the SF and PF properties are presented in Table 2.

**Table 2:** Physical properties of NFs.

Item	Fiber Type	
	Palm Fiber (PF)	Sisal Fiber (SF)
**Length (mm)**	10–30	10–30
**Tensile Strength (MPa)**	40–700	385–728
**Elongation of Break (%)**	8–18	2.75
**Diameter (mm)**	0.15–0.20	0.08–0.12
**Density (g/cm**^**3**^**)**	0.7–1.55	1.02–1.58
**Modulus of Elasticity (GPa)**	1–15	9–22

**Fig. 2:**
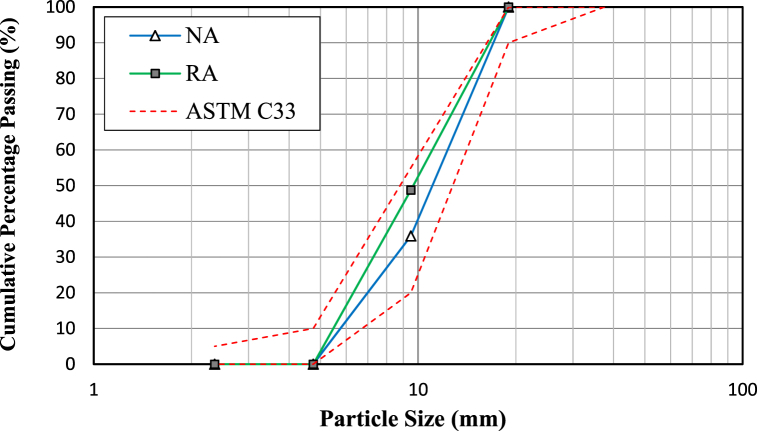
Particle size distributions of the NA and RA.

The natural concrete aggregate (NA) consisted of gravel that underwent continuous grading, whereas the recycled concrete aggregate (RA) was procured from a nearby facility in Bangkok, Thailand that recycles construction and demolition waste. Crushing and sieving are steps in the demolition of waste from the RA process. The physical properties of the coarse aggregates are listed in Table 3. The RA had a lower fineness modulus (5.96) than NA (6.22). For this experiment, RA was obtained from particles retained on a 4.75-mm sieve and passed through a 19-mm sieve. As shown in Fig. 2, the particle size distributions of the NA and RA were in accordance with the ASTM C33 standard [30]. The ADVA Cast 208 superplasticizer used in this study was produced by Grace Construction in Thailand. This is a composite polymer that requires high early strength and can achieve substantial water reduction. By working as a dispersing agent and reducing the water demand, the superplasticizer (polymer-based) ensures excellent workability and prevents the formation of agglomerates.

**Table 3:** Physical properties of the coarse aggregates.

Item	Aggregate Type	
	Recycled Aggregate (RA)	Natural Aggregate (NA)
**Particle size (mm)**	5–20	5–20
**Bulk density (kg.m-3)**	2460	2739
**Water absorption (%)**	5.67	1.3
**Mud content (%)**	2.4	0.7
**Crush value (%)**	17.4	9.9
**Acicular content (%)**	9.23	4.13
**Specific Gravity**	2.20	2.65
**Fineness Modulus**	5.96	6.22

### 2.2 Mix proportions

The RA replacement ratio and the PF, SF, and NS quantities were used as control variables to determine the effects of different amounts of these materials on the mechanical properties and microstructure of RAC. The volume content of the RA in the total coarse aggregate is denoted by the RA replacement ratio of 0 and 100 %. In this study, PFs and SFs were incorporated into the RAC at a dosage of 3 kg/m³, calculated as a percentage of the total weight of the concrete, to ensure consistent distribution throughout the mix [31]. Additionally, 3 % NS was replaced by cement, with the NS content defined as the mass ratio of NS to cement. To determine the effects of different amounts of PF, SF, and NS on the mechanical properties and microstructure of RAC, the above factors were used to produce fourteen different mixtures. The composition of the mixture was determined according to British Standard BS EN1260:2003 [32]. A water-to-binder ratio of 0.60 was employed, and an adequate amount of superplasticizer was utilized to add the desired properties of excellent workability to the concrete. The design specifications of these mixtures are listed in Table 4.

**Table 4:** Mix proportions of all concrete samples.

Sample	Mix proportion (kg.m-3)									
	Water	Cement	Sand	NA	RA	PF	SF	NS	Super P	Slump (mm)
**Control**	300	500	635	1050	0	0	0	0	10	123
**Recycle**	300	500	635	0	945	0	0	0	10	102
**CP**	300	500	635	1050	0	3	0	0	10	79
**RP**	300	500	635	0	945	3	0	0	10	77
**CS**	300	500	635	1050	0	0	3	0	10	70
**RS**	300	500	635	0	945	0	3	0	10	68
**CN**	300	485	635	1050	0	0	0	15	10	73
**RN**	300	485	635	0	945	0	0	15	10	72
**CPN**	300	485	635	1050	0	3	0	15	10	74
**RPN**	300	485	635	0	945	3	0	15	10	72
**CSN**	300	485	635	1050	0	0	3	15	10	66
**RSN**	300	485	635	0	945	0	3	15	10	65
**CPSN**	300	485	635	1050	0	3	3	25.5	10	62
**RPSN**	300	485	635	0	945	3	3	25.5	10	59

Remark: CP = Natural aggregate mixed Palm fiber.

RP = Recycled aggregate mixed Palm fiber.

CS = Natural aggregate mixed Sisal fiber.

RS = Recycled aggregate mixed Sisal fiber.

CN = Natural aggregate mixed nanosilica.

RN = Recycled aggregate mixed nanosilica.

CPN = Natural aggregate mixed Palm fiber and nanosilica.

RPN = Recycled aggregate mixed Palm fiber and nanosilica.

CSN = Natural aggregate mixed Sisal fiber and nanosilica.

RSN = Recycled aggregate mixed Sisal fiber and nanosilica.

CPSN = Natural aggregate mixed Palm and Sisal fiber with nanosilica.

RPSN = Recycled aggregate mixed Palm and Sisal fiber with nanosilica.

### 2.3 Sample preparation

Concrete containing NF and NS in RAC was prepared. Mixing procedures were performed in a rotary mixer according to the method presented in a previous paper [[33], [34], [35]]. The following methods were performed.

1. The RA was mixed with water at a normal blending rate for 2 min.
2. The NS particles were stirred into the remaining water at a rapid speed for 2 min at a regular speed.
3. The PF or SF was incorporated into the cement and mixed for 2 min at a regular rate.
4. With a steady motion, the sand was incorporated into the mixture from step 3 for 2 min.
5. The dry mixture from step (5) was stirred into the mixture from step (3), followed by the addition of the remaining water and total NA for 3 min.

To maintain the workability of the concrete despite the high water absorption properties of the recycled aggregates, a superplasticizer was added during the mixing process. This superplasticizer helps to reduce the water demand while keeping the concrete mixture workable. The slump was measured immediately after the preceding procedure. A slump test was performed to evaluate the workability and compressive strength of the concrete. Subsequently, the mixture was placed into molds and subjected to a 4-min vibration period on a vibrating table, followed by a demolding period of 24 h after casting. Two different specimens were created: a 100 × 100 × 100 mm cube and 150 × 150 × 500 mm prismatic beam. A mixed analysis was performed to determine how different concentrations of PF, SF, and NS affected the RAC microstructure after 28 d.

### 2.4 Test program

#### 2.4.1 Part I: investigate mechanical properties

Compressive strength is an essential characteristic required for most concrete applications. Generally, a high compressive strength indicates that the concrete is of high quality. All the concretes were tested for their compressive and flexural strengths in accordance with the ASTM C39 [36] and ASTM C78 [37] standards, respectively. Using the vibrator table, the concrete specimens were compacted into cubic blocks (100 × 100 × 100 mm) and prismatic beams (150 × 150 × 500 mm). After the concrete specimens were hardened in molds for 24 h following casting, they were cured in tap water at room temperature. Three concrete specimens were tested and used for each data point after 7 and 28 d.

#### 2.4.2 Part II: investigate microstructure

• Determination using scanning electron microscopy (SEM)

The cement concrete was crushed to a size of 10 × 10 × 10 mm, washed thoroughly with acetone to stop hydration, and vacuum dried at room temperature. A micrograph of the specimen was acquired using a Nova NanoSEM 450 instrument operating at an accelerating voltage of 15 kV and a current of 80 mA. A gold coating was applied to the specimens prior to observation.

• Determination using x-ray diffraction (XRD)

The X-ray diffractometer utilized to analyze the crystallite phases of 28-d-aged concrete was the D8000 equipped with CuK_α_ (k = 1.54056 A°) radiation at 40 mA and 40 kV. Data collection was performed in the 2θ range of 5–80°, with a step time of 0.1 s.

## 3 Results and discussion

### 3.1 Slump test

**Fig. 3:**
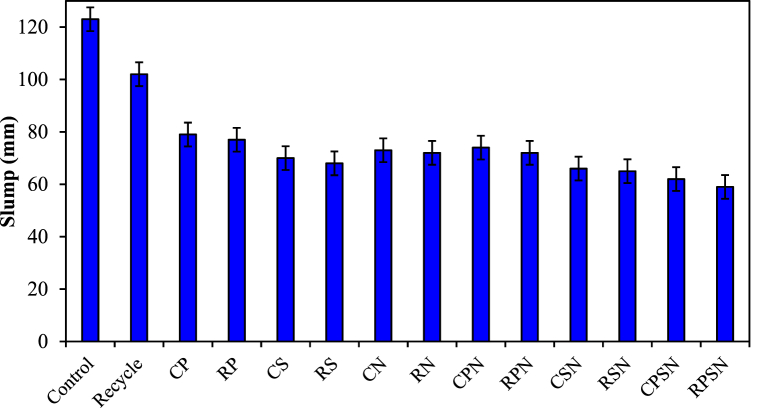
Measured values of fresh concrete slump test.

As one of the most significant indicators of concrete performance, the slump is directly correlated with the fluidity of the material. The values obtained from the slump test conducted on the RAC are presented in Table 4 and Fig. 3. The decrease diminished as the proportion of RA replacement, NF (PF and SF), and NS content increased. Mazlan and Awal [38] and Musa et al. [39] attributed this to the increased surface area and high water absorption of the fiber aggregates, which decreased the amount of water present in the mixture. A potential consequence of fiber water absorption is that the workability of the concrete mixture decreases with the duration of mixing and transport. The incorporation of aged mortar into RAC, which results in a rougher surface, is the primary determinant of the decreased workability of concrete [40,41]. Owing to the increased surface area, a larger quantity of cement slurry is necessary to attract PF and SF. Additionally, the network structure created by the dispersed fibers in concrete blocks its movement [42]. Because NS has a larger powder surface area and ultrafine particle size, it renders concrete mixtures less workable owing to the reduced amount of free water in wet blends [43]. Wang et al. (2019) [31] observed that the incorporation of NS into RAC affected its workability. This is because silica has a large specific surface area and nanoparticles, which make it easier for objects to stick together when van der Waals forces act on them [44]. An increase in NS to 8 % resulted in a further 35 % decrease in slump values, compared with the 15 % reduction observed in mixtures containing 3 % NS. In their study, Erdem et al. (2018) [45] explored the effects of colloidal NS supplemented with 1 % and 1.5 % on the workability of concrete composed entirely of RAC. The experiment demonstrated a decrease in the drop when the NS slurry was incorporated into the RAC mixture. It is feasible to conclude that the addition of NS to freshly prepared RAC mixtures results in a decline in their workability, with an additional reduction occurring as the quantity of cement replacement increases. This is mainly attributed to the small size and high specific surface area of NS, which necessitate a substantial quantity of water for uniform dispersion in saturated mixtures. The decline in RAC reinforced with NF (PF and SF) and NS diminishes as the RA replacement ratio and the contents of PF, SF, and NS increase.

### 3.2 Compressive strength

**Fig. 4:**
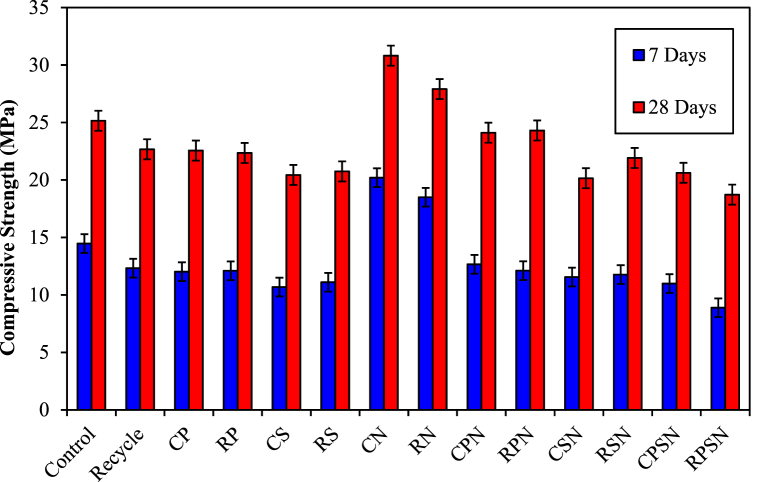
Compressive strengths of RAC after 7 and 28 d.

The increase in the RA replacement ratio resulted in an 11.1 % decrease in the compressive strength of RAC at 28 d compared with the control concrete, as illustrated in Fig. 4. These results agree with the conclusions obtained by Marie et al. (2012) [46]. The primary reason for this is that compared to the NA, the RA includes multiple interfaces [47]. The presence of a complex ITZ structure within RAC increases the number of defects, thereby increasing the probability of bonding failure when subjected to an extraneous force. Additionally, a greater number of microcracks in the RA following artificial secondary fragmentation serves as evidence that pressure occurs at the fracture tip [48,49]. The ITZ exhibits poor bonding performance between the old mortar and RA, which is vulnerable to bond slippage when subjected to loading [50,51]. The significance of these factors increases as the RA replacement ratio increases, resulting in a further decrease in compressive strength. The study revealed that confinement through transverse reinforcement significantly improved the mechanical properties of RAC, which is consistent with the finding that transverse reinforcement plays a critical role in enhancing RAC peak and ultimate strains, as demonstrated in similar studies of Munir et al. (2020) [52].

The primary purpose of incorporating NF (SF and PF) into concrete is to enhance its quality through the formation of an interconnected network of uniformly distributed fibers [53,54]. Compared to the control concrete without NF, the compressive strengths of RP and RS decreased by 11.1 % and 10.9 %, respectively, after 28 d compared with recycled concrete. The main reason is that an increase in the SF and PF contents results in a larger surface area of the fibers. Consequently, the density of concrete is diminished and internal defects escalate [53]. When subjected to a load, cracks rapidly develop stress concentrations, which result in both localized injury and spread toward surrounding areas [54]. Consequently, the crack and fracture contents increase as the SF and PF replacement ratios increase. Thus, an increase in the RA replacement ratio results in a decrease in the compressive strength of the RAC when the SF and PF contents are significant.

The compressive strength of concrete exhibited a further increase as the RA replacement ratio increased by 22.5 % and 11.0 % (CN and RN as control and recycled concrete), respectively) 28 d after the addition of NS. The compressive strength of concrete can be improved by adding sufficient NS [55]. Nazari and Riahi (2011) [56] found that when a sufficient amount of NS was added, the hydration products were C-S-H gels. Additionally, the filling of spaces within the concrete resulted in increased homogeneity and the compressive strength of the specimens increased. At a replacement ratio of 100 % RA for NS, the compressive strength increased proportionally with NS content. The main factor contributing to this was that diminished RA replacement ratios resulted in a comparatively lower porosity of the concrete. When NS particles adhere to the surface of the RA, they fill the microscopic holes within the RA. Because of the heightened effectiveness of the ITZ, an increase in its compressive strength was observed. Some NS particles are incapable of reacting with calcium hydroxide (CH) crystals when the NS concentration is high; instead, they leach directly as fillers [57]. As a result, an excessive NS content inhibits the enhancement of the compressive strength. Therefore, a higher quantity of NS leads to an increased probability of agglomeration, diminished workability, and a greater amount of entrapped air. These variables impede the complete use of high-activity energy in NS particles, leading to a reduction in the density of the mixture and, subsequently, its compressive strength. A higher RA replacement ratio results in the expansion of pores and holes within the concrete, requiring the addition of more NS particles to fill them. Consequently, an increase in the NS content results in a corresponding enhancement of the compressive strength at a 100 % RA replacement ratio. At 28 d, the compressive strength of the substance reinforced with NS and NF (SF and PF) decreased by 49.0 % (RN to RPSN). The main reason is that the RA contains a greater number of closed microcracks, which NS particles are unable to bridge effectively. An increase in the RA replacement ratio corresponds to a greater number of closed microcracks. Stress concentration quickly develops at the interface of cracks when subjected to loading, resulting in both localized injury and spread to the surrounding area [54]. The compressive strength results of the fiber-reinforced RAC in this study exhibit variability similar to that reported in previous research, highlighting the challenges and benefits of using fiber reinforcement in RAC [12].

### 3.3 Flexural strength

**Fig. 5:**
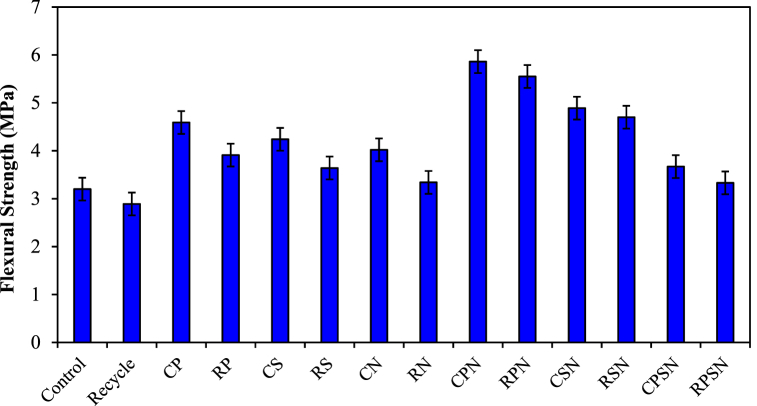
Flexural strength of RAC at 28 d.

Fig. 5 shows the results of the flexural strength tests after 28 d. With an increasing RA replacement ratio (recycled concrete), the flexural strength of the 28-d RAC decreased compared to that of the control concrete when RA was used alone. Singh et al. (2018) [58] and Allujami et al. (2022) [59] also identified this trend. A decrease in flexural strength occurred when the RA replacement was owing to the impact of the factors mentioned above. The flexural strengths of the alternative mixes decreased with increasing RA replacement ratios.

As shown in Fig. 5, PF and SF contributed significantly to the enhancement of flexural strength. The flexural strengths of RP and RS increased by 46.7 % and 26.0 %, respectively, compared with those of recycled concrete when the PF and SF contents were both 3 kg m^−3^. These findings are consistent with those reported by Lin et al. (2023) [11]. The limited diameters of PF and SF enabled them to efficiently prevent the growth of fractures. Consequently, the ITZ properties were improved by the application of PF and SF. From the above analysis, it can be concluded that PF and SF have a greater effect on the flexural strength of RAC than NS. NF increases flexural strength by inhibiting the formation of fractures. The load is promptly transferred to the fibers because of the interface between the fibers and the concrete components. The failure of fractures to fracture is due to the load-bearing propagation of cracks along the fibers. The combined resistance of the fibers and concrete matrix to force increases the flexural strength of the structure [60].

Samples devoid of fibers exhibited a moderate increase in flexural strength of 25.6 % with a 3 % increase in NS content (CN). Further addition of NS had no discernible effect on the flexural strength. However, the tensile strength characteristics of the specimens showed that as the NS content increased, the ability of the reinforcing fibers to induce flexural strength increased even more significantly. For example, the flexural strength of the mixtures containing the greatest amounts of PF and SF (CPN and CSN to CN) increased by 45.8 % and 38.1 %, respectively. The amount of NS added increased the weight of the cement by 3 % compared to the reference sample. The flexural strength tests revealed that NS, which has filler and pozzolanic properties, can improve the adhesion and structural properties of coarse cement matrix fibers and mortar aggregates [61]. Our study demonstrates that reinforcing RAC with natural fibers and NS significantly enhances its mechanical properties. However, when compared to the findings of Munir et al. (2020) [62], where the stress-strain performance was notably improved through steel spiral confinement, it suggests that combining these reinforcement methods could potentially yield even greater enhancements in the durability and strength of recycled concrete. The stress-strain behavior of RAC under uniaxial compression and tension observed in this study aligns with mesoscale modeling predictions, which highlight the complex interactions within the RAC microstructure under applied loads [63].

### 3.4 SEM analysis

**Fig. 6:**
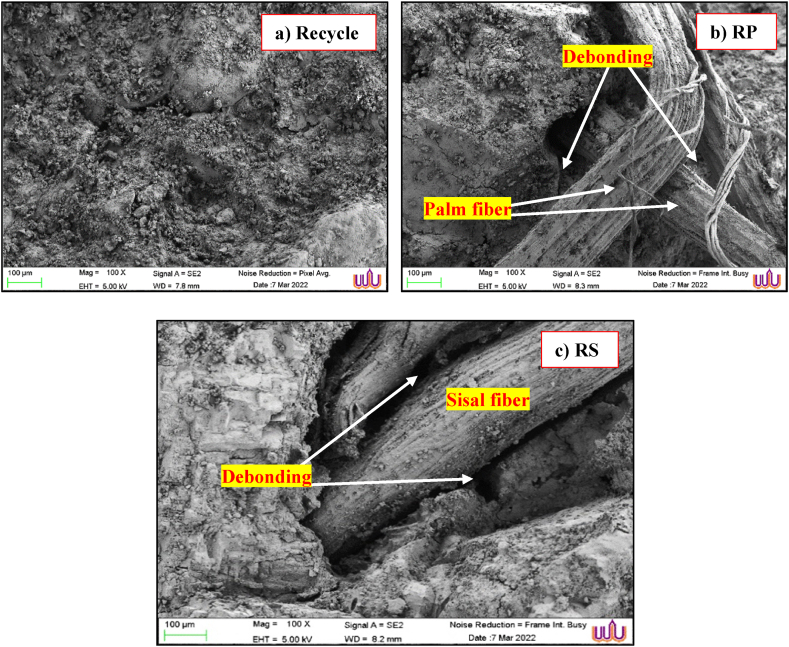
SEM images of RAC mixed with PF and SF after 28 d.

Fig. 6 shows the SEM images of RAC with NF and NS after 28 d. The SEM images reveal of the RAC as shown in Fig. 6a (Recycle), the surface texture, porosity, and the distribution of aggregates and binders can be observed. As illustrated in Fig. 6b and c (RP and RS), a weakened layer was discernible at the interface separating the cement matrix and the PF and SF. This indicates that the matrix was not properly bonded. This was responsible for the decrease in strength that occurred as the PF and SF increased. Ahmad et al. (2022) [64] also demonstrated this by showing an early age cement matrix and SF bond, followed by the appearance of a weak interfacial layer after 28 d. The performance integrity of the fibers is significantly influenced by the bond between the cement matrix and the PF and SF. The insufficient strength of the interfacial layer between the cement matrix PF and SF prevents the fibers from performing their functions as network supports. This weakens the RAC. As illustrated in Fig. 6b (RP), the PF was enveloped in cement paste. Thus, the condition of the fiber surface and the amount of alkali in the matrix would influence the bridging and bonding that occurs between the material and the fiber. Fine adhesion was observed between the fiber and matrix using SEM, with some debonding observed at the fiber end. Consequently, the increase in the flexural strength of the cement paste composite was prevented, and the fracture of the specimen was delayed. As indicated by the absence of matrix bonding in the SEM image in Fig. 6c (RS), the fibers failed to adhere effectively to the matrix when utilized as SF replacements. This explains why the strength decreased as the amount of added SFs increased. Similarly, Tunje et al. (2021) [65] identified SF as an effective bridging material for microcracks and as a means of enhancing the mechanical properties of concrete, as indicated by its enhanced performance and SEM images.

**Fig. 7:**
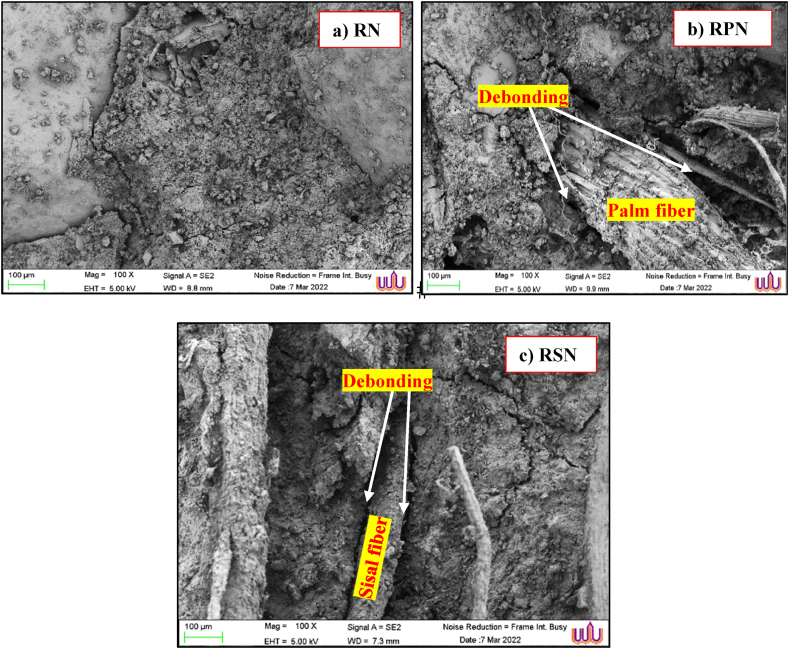
SEM image of RAC mixed with NS after 28 d.

The addition of NS improved the bonding properties, as illustrated in Fig. 7a–c. The C-S-H gel formed when NS chemically reacted with CH in the structure with PF and SF. This resulted in a decrease in the OH- concentration inside the matrix, leading to gel formation, as described by Khaloo et al. (2016) [66]. In the absence of NS, as shown in Fig. 7a (RN), the hydration product in RAC took the form of a plate-like crystal with a significant number of pores and a relatively loose structure. The connections became stronger, and the hydration products increased as NS increased. This phenomenon corresponds to the findings reported by Wengui et al. (2016) [67], indicating that the introduction of a suitable quantity of NS results in a reduction in pore size and content. In addition, as shown in Fig. 7b and c (RPN and RSN), adding an appropriate amount of NS to the RAC mixed with PF and SF made it more homogeneous, reduced the voids, and caused hydration products to build up in the matrix. Consequently, the incorporation of NS into the RAC may enhance its internal structure [68]. The result is a compact and homogeneous C-S-H structure in which the NS particles serve as the primary component and fill the voids within the concrete, resulting in a highly compact concrete matrix structure. In addition, the CH crystals that form when cement hydrates and interacts with water hold the NS particles together [69]. The limited space conditions make it difficult for all the CH crystals to form, which lowers the number of crystals, resulting in a reduction in the strengthening gel and an increase in the shrinkage creep of concrete [70]. Consequently, the structural integrity of the internal components of the concrete is compromised. Conversely, excessive NS may lead to particle agglomeration, which diminishes the particle surface area and dispersion. This subsequently blocks the ability of the particles to fully exploit their high activity energies. In addition to reducing the workability of concrete, the agglomeration of NS nanoparticles affects its durability.

### 3.5 XRD analysis

**Fig. 8:**
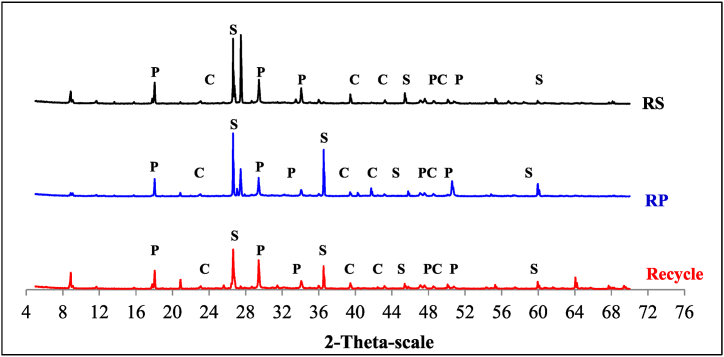
XRD patterns of RAC mixed with PF and SF after 28 d P: calcium hydroxide (CH); C: calcium carbonate (CC); S – silica dioxide (SiO_2_).

**Fig. 9:**
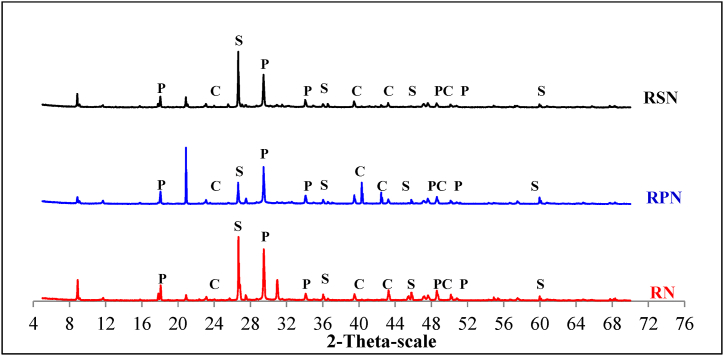
XRD patterns of RAC mixed with PF and SF after 28 d P: calcium hydroxide (CH); C: calcium carbonate (CC); S – silica dioxide (SiO_2_).

XRD to determine how the studied factors changed the hydration of the fiber–cement composites. This was achieved by examining the crystalline phases of the different types of NF (PF and SF) and NS in the RAC composites. After approximately 28 d, XRD patterns were observed for both the control sample (which did not contain NF or NS) and the composites that contained NF and NS, as shown in Fig. 8, Fig. 9. However, changes in the formulated composite type and fiber content affected the intensity of the peaks, particularly those of portlandite (P), calcite (C), and quartz (Q), indicating that CH, calcium carbonate (CC), and silica dioxide (SiO_2_) were present in the mixture.

By comparing the diffractograms obtained in this study with those reported in the literature [71,72], it was confirmed that the crystalline phases formed during the hydration of the different fiber–cement composites and the control sample were comparable (Fig. 8). This figure shows how the peak intensity changed at 2θ (theta) angles of 18.08°, 34.08°, 47.12°, and 50.79°, which are related to the properties of CH or portlandite (P) [65]. In the fiber–cement composites, the intensities of CH, CC, and SiO_2_ varied according to the types and content of the NF utilized. In composites containing NF, an increase in the fiber content resulted in a decrease in the CH content and an increase in the CC and SiO_2_ contents.

To examine the impact of NS on cement hydration, XRD analysis was performed on the RAC, as illustrated in Fig. 9. After the addition of NS, the strength of the XRD peak and its intensity related to the CH crystals decreased. Furthermore, the main crystal pattern of the concrete was that of CH crystals, suggesting that NS effectively obstructed the development of CH crystals. Owing to the low crystallinity of the C-S-H gel, the characteristic peak in the XRD analysis was small. One theory states that the NS on the surface of the changed NF may have made it easier for the fibers to interact with the cement matrix by acting as a starting point. In this way of thinking, studies by Andrade et al. (2018) [73] and Qing et al. (2007) [2] found that cement pastes with NS had weaker CH peaks than reference pastes. Furthermore, cement pastes containing NS exhibited greater compressive strengths than those lacking NS in both investigations. The reaction kinetics of Portland cement were affected by the ability of silica (mainly colloidal silica, microsilica, and NS) to react with the cementitious matrix. As a result, the concentration of CH decreased, and some properties of cementitious pastes and composites improved after drying [[74], [75], [76]]. An additional significant factor pertains to the decrease in pH that occurs with a reduction in the CH concentration [77]. This can be highly advantageous for composites reinforced with NF given that NFs have shorter lifespans in alkaline environments. Finally, the cement performances of the modified NS and NF in RAC were superior to those of the control sample. As previously noted, these enhancements were primarily attributable to the silica nanoparticles present on the NF surface. This is because they increased both the specific surface area and surface irregularity, thereby enhancing the bond interaction with the cement matrix. NF cellulose fiber is less likely to absorb water, which means that it will not expand and contract unevenly during weathering. This indicates that it will remain mixed in the cement matrix during the molding process [78]. When NFs in fiber–cement composites stick together, weak and open spaces called voids and porous regions can appear, which weaken the composites and make them less effective [79].

## 4 Conclusion

1. The incorporation of NS and NF into RAC led to a reduction in workability, evidenced by a decrease in slump values as NS and NF content increased.
2. The flexural strength of RAC was primarily influenced by the NF content, with NS showing minimal impact. The optimal flexural strength was observed at a 100 % recycled aggregate replacement when combined with both NF and NS.
3. While the addition of NS initially improved the compressive and flexural strengths due to pozzolanic reactions, a higher NF content caused a decline in these properties due to increased voids and dispersion challenges.
4. NS-modified NF effectively enhanced the mechanical properties of RAC by bridging microcracks, as confirmed by SEM analysis. The addition of NS improved the cement matrix microstructure, increasing its density, reducing pore size, and strengthening the ITZ.
5. XRD analysis revealed that NS significantly reduced calcium hydroxide content in the cement matrix, highlighting its critical role in enhancing the pozzolanic activity of the concrete.

## CRediT authorship contribution statement

**Sattawat Haruehansapong:** Software, Methodology, Investigation, Formal analysis, Conceptualization. **Prachoom Khamput:** Writing – original draft, Supervision, Data curation. **Pruchaya Yoddumrong:** Visualization, Investigation. **Wunchock Kroehong:** Visualization, Investigation. **Vichayaphong Thuadao:** Resources, Data curation. **Akkadath Abdulmatin:** Writing – review & editing. **Wachirakorn Senawang:** Writing – review & editing. **Tawich Pulngern:** Writing – review & editing, Supervision.

## Informed consent

For this type of study, formal consent is not required.

## Data availability

Since no datasets were created or analyzed for this topic, data sharing is not applicable.

## Additional information

No additional information is available for this paper.

## Ethical approval

Not Applicable.

## Funding statement

This research did not receive any official grant from funding agencies in the public, commercial, or not-for-profit sectors.

## Declaration of competing interest

The authors declare that they have no known competing financial interests or personal relationships that could have appeared to influence the work reported in this paper.
